# The complete mitochondrial genome of Marsh Sandpiper *Tringa stagnatilis* (Charadriiformes, Scolopacidae)

**DOI:** 10.1080/23802359.2020.1787895

**Published:** 2020-07-11

**Authors:** Jingjing Ding, Hao Wang, Deyun Tai, Xue Xu, Chaochao Hu, Qing Chang

**Affiliations:** a Jiangsu Academy of Forestry, Nanjing, China; b College of Life Sciences, Jiangsu Key Laboratory for Biodiversity and Biotechnology, Nanjing Normal University, Nanjing, China; c Analytical and Testing Center, Nanjing Normal University, Nanjing, China

**Keywords:** Mitogenome, Charadriiformes, *Tringa stagnatilis*

## Abstract

The complete mitochondrial genome of Marsh Sandpiper *Tringa stagnatilis* was sequenced in this study. The circular mitogenome was 16,799 bp in length, which contained 13 protein-coding genes, two ribosomal RNAs (12S rRNA and 16S rRNA), 22 transfer RNA genes, and a D-loop region. The overall nucleotide composition was A: 31.51%, T: 25.45%, C: 29.51%, and G: 13.53%. Twenty-eight genes were encoded on the heavy strand, and the remaining nine genes were encoded on the light strand. The common start codon was ATG, and four stop codons and an incomplete stop codon (T–) were used in PCGs. This study improves our understanding of the mitogenomic characteristics and its phylogenetic relationships within Charadriiformes.

The Marsh Sandpiper *Tringa stagnatilis* is a small wader, with a long bill and yellowish legs. This species is a full migrant, traveling overland on a broad front between its breeding grounds in central Asia, and its wintering grounds in sub-Saharan Africa, southern Asia, Indonesia, and Australia. Despite the fact that the population trend appears to be decreasing, it is evaluated as Least Concern (BirdLife International 2016). Nevertheless, the basic genetics data of *T. stagnatilis* is still unclear. In this study, we sequenced the complete mitogenome of *T. stagnatilis* to further understanding the mitogenomic characteristics and its phylogenetic relationships within Charadriiformes.

The specimen of *T. stagnatilis* was collected from a derelict and abandoned mist net in Xiaoyangkou, Nantong City, Jiangsu Province, China (32°33′38.84″ N, 121°10′08.83″ E). After collection, the tissue was initially preserved in 95% ethanol, and then transferred to −20 °C in laboratory for long-term storage at Nanjing Normal University (specimen voucher: NJNU-Tsta06). Total genomic DNA was extracted with standard phenol–chloroform methods (Sambrook et al. 1989). The sequencing libraries with average insert sizes of approximately 300 bp were prepared, and then sequenced as 150 bp paired-end runs (about 15 Gb raw data) on the Illumina HiSeq 2000 platform (Illumina, San Diego, CA). The sequence quality analysis, data trimming, and *de novo* assembling were used by the software of Geneious 9.1.4 (Kearse et al. 2012). The sequence annotation was preliminarily defined by MITOS web server and identified by alignment with other mitogenome of Charadriiformes species (Bernt et al. 2013).

The circular mitogenomes is 16,799 bp (GenBank: MT572847) in length, consisting of 13 protein-coding genes, 2 ribosomal RNAs (12S rRNA and 16S rRNA), 22 transfer RNA genes, and a non-coding region, and all the genes are identified, without showing any structural rearrangement. The genes of ND6 and eight tRNA (tRNA^Gln^, tRNA^Ala^, tRNA^Asn^, tRNA^Cys^, tRNA^Tyr^, tRNA^Ser^, tRNA^Pro^, and tRNA^Glu^) are encoded on the light strand, whereas the other genes are located on the heavy strand. The overall nucleotide composition was A: 31.51%, T: 25.45%, C: 29.51%, and G: 13.53%. The total length of 13 protein coding genes is 11,397 bp accounting for 67.84% of the complete genome. The start codon ATG appeared in 11 PCGs, with the exception of COI (GTG) and ND3 (ATA). The use of stop codons is more diverse, comparatively. Four stop codons (TAA, TAG, AGG, and AGA) and an incomplete stop codon (T––) were used.

Phylogenetic reconstruction was performed using the maximum likelihood (ML) method with the software of MEGA X (Kumar et al. 2018). The dataset of nearly complete mitogenome (15,239 bp) of 14 Charadriiformes species was used, and two species were used (*Phodilus badius*, *Gallus gallu*s) as outgroups. ML phylogeny was inferred under the GTR + G+I model for 1000 bootstraps (Kumar et al. 2018). The phylogenetic analysis (Figure 1) resolved great mitochondrial divergence within the Charadriiformes. This study improves our understanding of the evolution of mitogenome in Charadriiformes.

**Figure 1.**
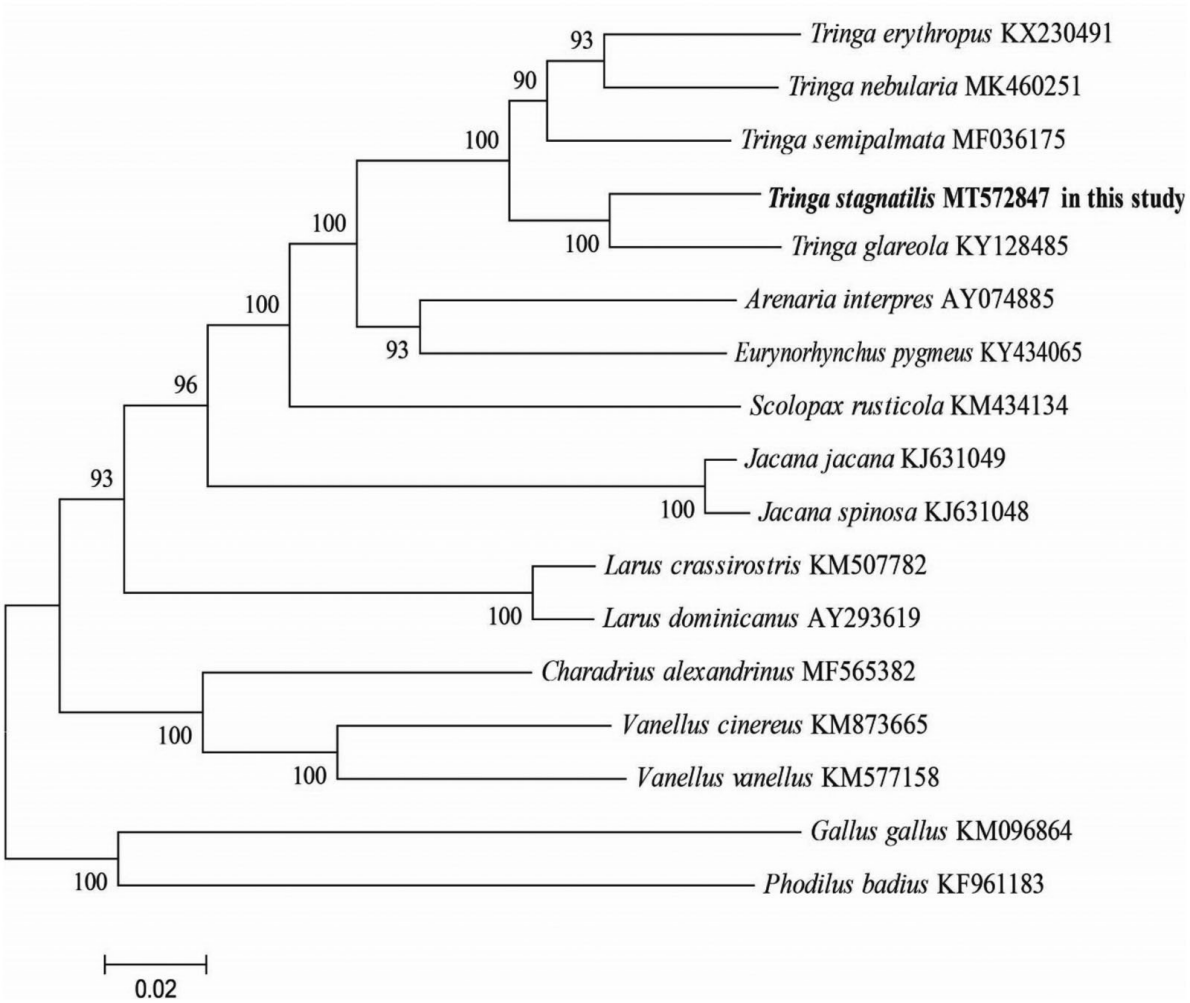
Phylogeny of *T. stagnatilis* and closely related 14 mitochondrial sequences constructed using the maximum likelihood (ML) method by analyzing mitochondrial complete genome. Numbers above each branches are the ML bootstrap support.

## Data Availability

The data that support the findings of this study are openly available in National Center for Biotechnology Information (NCBI) at https://www.ncbi.nlm.nih.gov/, reference number MT572847.
